# Birth Weight, Intrauterine Growth Retardation and Fetal Susceptibility to Porcine Reproductive and Respiratory Syndrome Virus

**DOI:** 10.1371/journal.pone.0109541

**Published:** 2014-10-02

**Authors:** Andrea Ladinig, George Foxcroft, Carolyn Ashley, Joan K. Lunney, Graham Plastow, John C. S. Harding

**Affiliations:** 1 Department of Large Animal Clinical Sciences, Western College of Veterinary Medicine, University of Saskatchewan, Saskatoon, Saskatchewan, Canada; 2 Department of Agricultural, Food, and Nutritional Science, Faculty of Agricultural, Life and Environmental Sciences, University of Alberta, Edmonton, Alberta, Canada; 3 Animal Parasitic Diseases Laboratory, Beltsville Agricultural Research Center, Agricultural Research Service, United States Department of Agriculture, Beltsville, Maryland, United States of America; Virginia Polytechnic Institute and State University, United States of America

## Abstract

The severity of porcine reproductive and respiratory syndrome was compared in pregnant gilts originating from high and low birth weight litters. One-hundred and eleven pregnant gilts experimentally infected with porcine reproductive and respiratory syndrome virus on gestation day 85 (±1) were necropsied along with their fetuses 21 days later. Ovulation rates and litter size did not differ between groups, but fetuses from low birth weight gilts were shorter, lighter and demonstrated evidence of asymmetric growth with large brain:organ weight ratios (i.e. brain sparing). The number of intrauterine growth retarded fetuses, defined by brain:organ weight ratios greater than 1 standard deviation from the mean, was significantly greater in low, compared to high, birth weight gilts. Although γδ T cells significantly decreased over time in high compared to low birth weight gilts, viral load in serum and tissues, gilt serum cytokine levels, and litter outcome, including the percent dead fetuses per litter, did not differ by birth weight group. Thus, this study provided no substantive evidence that the severity of porcine reproductive and respiratory syndrome is affected by dam birth weight. However, intrauterine growth retarded fetuses had lower viral loads in both fetal thymus and in endometrium adjacent to the umbilical stump. Crown rump length did not significantly differ between fetuses that survived and those that died at least one week prior to termination. Taken together, this study clearly demonstrates that birth weight is a transgenerational trait in pigs, and provides evidence that larger fetuses are more susceptible to transplacental PRRSv infection.

## Introduction

The number of offspring is an important economic trait in livestock species [1]. Due to the selection of highly prolific sows, the number of piglets born per litter has increased considerably over the last decade. However, selection for increased litter size can increase the risk of intrauterine growth retardation (IUGR), which is defined as impaired growth and development of embryos and fetuses and their organs characterized by disproportionate growth of brain compared to other fetal organs (relative brain sparing) [2], [3]. Multiple genetic, epigenetic, endocrine and environmental factors regulate fetal growth and may therefore contribute to IUGR, which can be assessed by the measurement of fetal birth weight (BW) [3]. The transgenerational nature of BW was established in humans by demonstrating that low BW mothers deliver lower BW babies [4], [5]. BW in pigs is related to litter size and is responsive to genetic selection [1], [6]. Pigs exhibit the most severe, naturally occurring IUGR amongst domestic animals [3]. Naturally occurring IUGR results in asymmetrical fetal growth, typified by relative sparing of the brain compared to all other fetal organs [2], [7].

The postnatal consequences of IUGR are diverse. In various livestock species, IUGR is associated with high neonatal morbidity and mortality [8], abnormal gastrointestinal morphologies and dysfunction [9], [10], altered carcass composition and development of muscle fibers [7], [11], and decreased reproductive performance [12], [13]. In humans, IUGR is associated with increased risk of infectious disease mortality [14]–[16], decreased thymic function [17], impaired cell-mediated immunity [18] and impaired humoral immune responses following typhoid vaccination [19], [20]. The negative effects of prenatal maternal stress on postnatal immune responses have been studied in pigs, with effects on serum immunoglobulin G concentrations in suckling piglets, lymphocyte proliferation responses to mitogens, proinflammatory cytokine and neuroendocrine hormone production have been documented [21]–[24]. However, the characterization of immune responses and disease susceptibility in low BW or IUGR pigs is incomplete. To our knowledge, no studies have evaluated this relationship using porcine reproductive and respiratory syndrome (PRRS), one of the most economically important diseases worldwide in the swine industry.

We have recently completed a large scale project with the goal of investigating phenotypic and genotypic predictors of reproductive PRRS severity [25]. In this experiment, a virulent strain of PRRS virus (PRRSv) was used to inoculate pregnant gilts at gestation day 85. Animals were euthanized and necropsied 21 days post inoculation (dpi), approximately one week prior to normal term. Numerous phenotypic responses were characterized, including virus levels in serum and tissues, changes in leukocyte subsets and cytokine proteins over time in gilt blood, and fetal preservation and mortality. Also evaluated were detailed measurements of the position, size and weight of fetuses and their internal organs. For this experiment, dams were specifically selected from high and low BW litters in order to determine if the BW of the dam influences PRRS severity.

The objectives of this present study were to: 1) determine the transgenerational consequences of BW by comparing litter size, ovarian and fetal characteristics between gilts born from low and high BW litters; 2) compare immunologic responses and PRRS severity between low and high BW gilts in a reproductive model; 3) investigate relationships between IUGR and PRRS severity in fetuses.

## Materials and Methods

### Ethics Statement

Inoculation of gilts or sows in the last trimester of gestation is a widely accepted and commonly used model for studying reproductive PRRS [26]–[31]. Although we recognize that some fetuses die after inoculation, no alternative models are available to study the reproductive effects of PRRSv infection. Fetal death can occur any time after inoculation and it is not possible to predict if and when it will occur in any individual fetus. Monitoring fetal stress and discomfort is not feasible in a litter bearing species like swine. A humane intervention point (HIP) checklist was developed and approved specifically for this project. Gilts were monitored according to the HIP checklist twice daily. Clinical signs in gilts were mild or absent, so medical interventions such as analgesics or anesthetics were not justified. Animal numbers were carefully considered and the number of inoculated gilts was selected to enable both deep phenotyping and genotyping of gilts and fetuses. Given that fetal death was an outcome, the experimental protocol was considered carefully before approval by the University of Saskatchewan's Animal Research Ethics Board. It adhered to the Canadian Council on Animal Care guidelines for humane animal use (permit #20110102).

### Animal experiment and sample collection

The experimental protocol has been described in detail [25] and a few additional salient points follow. In a high-health nucleus herd, purebred Landrace gilts were selected at approximately 150 days of age in bi-weekly repetitions according to their BW. For this, the average litter BW was compared to the historical average litter BW of farm cohorts after controlling for total born litter size and parity. A Z-score was calculated for each litter according to the formula Z = (*x* - *m*/σ) where *x* is average litter BW of the litter to be standardized and *m* and σ are the mean and SD of the appropriate cohort population. Fifty-four low and 57 high BW litters were identified as having a Z-score greater or less than 0.7. Where possible, the selected litters were from multiparous sows that had previously farrowed one or more high or low BW litters. Preference was also given to litters in which the sow's average litter BW was above a Z-score of +0.7 or below a Z-score of −0.7. One gilt (hereafter referred to as "gilt" or "dam") with a BW closest to the average for the litter was selected. Estrus was synchronized in successive cohorts of gilts which were then bred homospermically to Yorkshire boars (3 billion motile spermatozoa per dose). In total 24 boars were used and no more than 6 gilts (blocked, 3 low and 3 high BW) were bred with semen from one boar. Pregnant gilts were housed in stalls until gestation day 80 (±1) then transported to a biosafety level 2 animal care facility at the University of Saskatchewan. On experimental day 0 (D0), the pregnant gilts (gestation day 85 ±1) were inoculated with 1×10^5^ TCID_50_ PRRSv isolate NVSL 97-7895 (50% intranasal, 50% intramuscular). Gilts were monitored daily for clinical signs and demeanour, including the assessment of rectal temperature and feed intake. Serum and whole blood samples were collected on D0, D2, D6, D19 and D21. On D21, the gilts were humanely euthanized and necropsy examinations performed on gilts and their fetuses. The weight of both ovaries and the number and weight of corpora lutea (CL) were recorded for each gilt. Fetal preservation status was categorized as: viable (VIA), meconium stained (MEC), decomposed (DEC; dead with primarily white skin) and autolyzed (AUT; dead with over 50% brown discoloured skin) [25]. The weight, crown rump length (CRL) and sex of each fetus were recorded. The fetal brain, liver, lung, heart, spleen and kidney were removed and weighed. Portions of reproductive lymph node (*Lnn. uterini*), lung, tracheobronchial lymph node, and tonsil were collected from each gilt and stored at -80°C until further processing. Fetal thymus as well as a piece of endometrium (including adherent placental layers) adjacent to the umbilical stump of each fetus were immediately frozen at −80°C until further processing.

### Quantification of PRRSv RNA

PRRSv RNA concentrations were measured in gilt serum (D0, D2, D6, D21) and gilt and fetal tissue samples collected at termination using a strain-specific in-house quantitative reverse transcription polymerase chain reaction (qRT-PCR). Primers were designed to target a highly conserved region at the C-terminal end of ORF7 of NVSL 97-7895 [25]. Results were reported as logarithm base 10 target RNA concentration per mg or µL. PRRSv viral load (VL) in fetal thymus and endometrium were categorized as: negative (NEG) = below detection limit of the qRT-PCR; low (LVL) = positive, non-quantifiable as below lowest standard; medium (MVL) = positive, quantifiable and lower than mean of quantifiable samples, or high (HVL) = positive, quantifiable and higher than mean of quantifiable samples.

### FCM analyses of PBMC

Automated white blood cell (WBC) counts (Z2 Coulter Particle Count and Size Analyzer, Beckman Coulter Inc., FL, US) and manual differential counts were performed (300 cells total) on heparinized blood samples collected on D0, D2, D6 and D19. Peripheral blood mononuclear cells (PBMC) were isolated by gradient centrifugation (Ficoll-Paque PLUS, GE Healthcare, Mississauga, ON) and analysed phenotypically by flow cytometry (FCM). Major PBMC populations were defined as follows: myeloid cells (CD172a^+^), NK cells (CD8α^+^CD3^-^), B cells (CD79α^+^), and T cells (CD3^+^). In addition, three distinct subpopulations of T cells were identified and analyzed: γδ T cells (T cell receptor γδ^+^), T helper cells (CD3^+^CD4^+^), and cytolytic T cells (CTLs) (CD3^+^CD8β^+^). Absolute numbers of different cell subsets were calculated using results from automated WBC and differential counts (total number of lymphocytes plus total number of monocytes).

### Cytokine testing

Serum samples collected on D0, D2, D6, and D21 were analysed for the following innate, T helper 1 (Th1), T helper 2 (Th2), and regulatory cytokines/chemokines by Fluorescent Microsphere Immunoassays (FMIA): interleukin (IL) 1β, IL4, IL8, IL10, IL12, chemokine ligand 2 (CCL2), and interferon alpha (IFNα). A 7-plex in-house FMIA assay was developed as previously described with several modifications [32]: magnetic rather than polystyrene beads were used; serum from a healthy, non-infected gilt with no measurable levels of relevant cytokines by FMIA was used as negative control and to prepare serial dilutions of cytokine standards. An enzyme-linked immunosorbent assay (ELISA) (Novex Swine IFNγ antibody duoset kit, Life Technologies Inc., Burlington, ON) was used according to the manufacturer's instructions to measure IFNγ levels in serum.

### Statistical analysis

All statistical analyses were performed using Stata v13 (StataCorp, College Station, TX). Unless otherwise noted, multilevel, mixed-effects regression models were used whereby gilt-level variables accounted for experimental replicate as a random effect, and fetal-level variables accounted for dam of origin as a random effect. Initially, sire ID was included as a random effect in all appropriate models, but since the proportion of the total variance attributed to sire was negligible (ranged from only 0 to 1% depending on the model), sire was removed from the final models for parsimony. Continuous variables were evaluated using two-level linear regression (XTMIXED) and count variables using two-level Poisson regression (MEPOISSON). Full models were developed by including biologically plausible predictor variables of the outcome of interest if *P*<0.2 in univariate analyses. This was followed by a stepwise backward removal of predictors with the highest *P* value. Parsimonious, final models contained only predictor variable(s) for which *P*<0.05. All final models were evaluated to ensure normality and homoscedasticity of residuals. Analyses of fetal or organ weights only included data from live fetuses because autolysis precluded the accurate measurement of weight of the DEC and AUT fetuses.

#### Objective 1

To assess the potential transgenerational consequences of dam BW, litter characteristics (number of fetuses), ovarian characteristics (ovary weight, CL counts and weights), and fetal morphometrics (weights, CRL, brain:organ weight ratios) were compared between high and low BW dams. Additionally, the numbers of IUGR and non-IUGR fetuses were compared between low and high BW gilts using a Pearson's chi^2^ test. IUGR fetuses were defined as those with brain:organ weight ratios greater than +1 SD from the mean. Conversely non-IUGR fetuses were those with brain:organ weight ratios less than -1 SD from the mean. Collectively, these represented the top 16% and bottom 16% of the fetal population, respectively. Brain:organ weight ratios were calculated across a spectrum of fetal organs (liver, lung, heart, spleen, kidney) but only for live (VIA and MEC) fetuses.

#### Objective 2

To evaluate the potential effect of dam BW on PRRS severity, dichotomous variables including the presence or absence of fever and reduced feed intake post inoculation were evaluated using a Fisher's exact test. Other variables characterizing PRRS severity in low and high BW gilts included relevant gilt-level (percent dead fetuses per litter, number of fetuses per litter in each preservation category, viral load in gilt serum over time (area under the curve (AUC) from 0 to 21 dpi) and tissues, AUC for PBMC subsets and serum cytokines), and fetal-level (viral load in fetal thymus and endometrium, body weights of VIA and MEC live fetuses) outcomes.

#### Objective 3

Multiple approaches were used to determine potential relationships between IUGR and PRRSv severity. Firstly, a proportional odds model determined the probability that a fetus, based on its brain:organ ratio, would fall into one of four semi-quantitative viral load categories described above. The second approach compared viral load between IUGR and non-IUGR fetuses. The third approach compared CRL across fetal preservation category to determine if dead fetuses, particularly AUT, were significantly shorter than live fetuses.

## Results

### Effects of PRRSv infection on dam and fetal health

Detailed results of the PRRSv infection model are described in Ladinig *et al.* [25]. Briefly, all challenged gilts were viremic by 2 dpi and fetal mortality was 41 ±22.8% in PRRSv infected litters. Levels of PRRSv RNA were highest and most persistent in uterine and tracheobronchial lymph node of gilts. In fetuses, the highest viral load was detected in MEC and declined in DEC and AUT fetuses. The number of fetuses falling into the four viral load categories based on PRRSv RNA levels in fetal thymus and endometrium, for each fetal preservation category and combined, are presented in Figure S1. Pearson's correlation coefficient for PRRS RNA concentration in fetal thymus and endometrium collected adjacent to the umbilical stump was 0.73.

### Transgenerational consequences of dam BW

The average litter BW of the litters in which the low and high BW gilts were born, and the BW of selected gilts were significantly lower in low compared to high BW gilts (Table 1). Eighteen of 54 low BW and 26/57 high BW gilts were born from litters in which the sow's BW was above or below a Z-score of 0.7. Twenty sows that provided low BW gilts had repeatedly farrowed low BW litters (range 1–3 previous low BW litters), while consecutive high BW litters were recorded for 37 sows that provided high BW gilts (range 1–5 previous high BW litters).

**Table 1:** Characteristics of litters from which the high and low gilts were selected and reproductive characteristics of the selected low and high BW gilts used in the study.

	Low BW (n = 54)	High BW (n = 57)	*P* value
***Characteristics of litters of origin***			
Average litter BW dam (kg)	1.2 (0.2)	1.7 (0.2)	<0.001
BW of selected gilts (kg)	1.3 (0.2)	1.7 (0.2)	<0.001
Dam litter size	13.1 (2.6)	14.0 (2.5)	0.075
Z-score	−1.3 (0.5)	1.3 (0.5)	<0.001
Average parity dam	2.3 (1.5)	2.8 (1.5)	0.11
***Characteristics of low and high BW gilts***			
CL counts	22.3 (9.9)	21.1 (7.4)	0.46
Weight ovaries (g)	25.7 (9.8)	26.0 (7.6)	0.88
Weight CL (g)	16.8 (6.3)	16.5 (5.4)	0.71
Total litter size	12.6 (3.6)	13.1 (4.1)	0.47

The following characteristics of litters used for gilt selection are presented: average litter birth weight (BW) in which selected gilts were born (kg), the mean BW of selected gilts (kg), the mean litter size and average parity of dams, as well as calculated Z-scores controlling for total born litter size and parity. Reproductive characteristics of low and high BW gilts are presented as: mean ovulation rate (counts of corpora lutea (CL)), mean ovarian weights and summed weights of all CLs (g) and mean litter size including alive, dead and mummified (inspissated fetuses with CRL <20 cm) fetuses.

Ovulation rates, total litter size, ovarian weights and summed weights of all CLs did not significantly differ between low and high BW gilts (Table 1).

Live (VIA, MEC) fetuses of low BW gilts, however, were shorter, lighter and evidenced brain sparing. The weights of all investigated fetal organs, with the exception of brain, were significantly lower in fetuses from low compared to high BW gilts (Table 2). Fetal weight was significantly associated with sex and total litter size. Male fetuses were on average 41 g heavier than female fetuses, and for each unit increase in litter size, fetal weight decreased approximately 30 g (Table 2). All brain:organ weight ratios were significantly greater in fetuses of low BW gilts, except the brain:kidney weight ratio, which showed a trend in the same direction (*P* = 0.052) (Table 2). The number of IUGR fetuses, those with extreme brain:organ weight ratios, was significantly greater in low compared to high BW gilts (Table 3). These results were consistent across all brain:organ weight ratios, with odds ratios ranging from 2.6 to 4.2 depending on the organ.

**Table 2:** Comparison of fetal weight and morphometrics in low and high BW gilts following PRRSv inoculation.

	Mean (SD)		*P* (β)				
	low BW (n = 415)	high BW (n = 407)	LoHi BW	Sex	LS	Preservation	VL thymus
weight fetus	936.2 (249.4)	1034.3 (252.6)	<0.001 (106.1)	0.003 (41.4)	<0.001 (−29.8)	ns	ns
weight brain	26.0 (2.8)	26.5 (2.8)	0.058 (0.6)	0.009 (0.4)	0.004 (−0.1)	<0.001 (−2.0)	<0.001 (−0.1)
weight liver	24.5 (8.9)	28.3 (9.1)	0.002 (3.1)	0.001 (1.6)	<0.001 (−0.9)	<0.001 (6.7)	0.006 (0.2)
weight lung	27.0 (8.2)	29.9 (8.9)	<0.001 (3.3)	0.013 (1.2)	<0.001 (−0.8)	ns	<0.001 (−0.6)
weight heart	7.6 (2.1)	8.4 (2.1)	0.001 (0.8)	0.004 (0.3)	<0.001 (−0.2)	ns	ns
weight spleen	1.5 (0.6)	1.6 (0.6)	0.018 (0.2)	0.001 (0.1)	<0.001 (−0.1)	0.002 (0.2)	<0.001 (0.04)
weight kidney	9.0 (3.0)	10.2 (3.5)	0.005 (1.1)	ns	<0.001 (−0.3)	ns	<0.001 (0.3)
brain:liver	1.2 (0.4)	1.0 (0.4)	0.001 (−0.1)	0.037 (−0.05)	<0.001 (0.04)	<0.001 (−0.3)	0.013 (−0.01)
brain:lung	1.1 (0.3)	1.0 (0.3)	0.002 (−0.1)	ns	<0.001 (0.03)	ns	<0.001 (0.01)
brain:heart	3.7 (1.0)	3.3 (0.9)	0.002 (−0.3)	ns	<0.001 (0.1)	<0.001 (−0.3)	0.005 (−0.03)
brain:spleen	20.5 (8.0)	18.6 (7.8)	0.034 (−1.7)	0.025 (−1.0)	<0.001 (0.7)	<0.001 (−2.7)	<0.001 (−0.5)
brain:kidney	3.2 (1.2)	2.9 (1.5)	0.052 (−0.2)	ns	<0.001 (0.1)	0.005 (−0.4)	<0.001 (−0.1)
CRL	28.0 (2.6)	28.8 (2.6)	0.001 (−0.9)	ns	<0.001 (−0.2)	0.001 (−0.7)	ns

*Left columns:* Mean (SD) fetal and fetal organ weights (g) of live fetuses (VIA, MEC) at termination, 21 days post-inoculation (gestation day 106±1) are presented for low and high birth weight (BW) gilts. Additionally, mean brain:organ weight ratios (SD) and crown-rump-length (CRL, cm) are displayed. *Right columns: P* values and beta coefficients (β) obtained by two-level, linear, mixed-effects regression models are presented for fixed effects retained in the parsimonious (final) model. β represents the difference between predictor categories or per unit increase in continuous variables. LoHi BW: 0 = low BW dam, 1 = high BW dam; Sex: 0 = female, 1 = male; LS: effect of a unit increase in litter size (fetal number); Preservation: fetal preservation at termination 0 = viable, 1 = meconium stained; VL thymus = effect of a unit increase in PRRSv RNA concentration (log_10_ target copies/mg) in fetal thymus collected at termination; ns = not significant (*P*>0.05).

**Table 3:** Number of IUGR fetuses in low and high BW gilts.

		low BW	high BW	Pearson chi^2^	Odds ratio
brain:liver	non-IUGR	46	85	*P*<0.001	4.2
	IUGR	91	40		
brain:lung	non-IUGR	51	80	*P*<0.001	2.6
	IUGR	82	49		
brain:heart	non-IUGR	52	79	*P*<0.001	2.7
	IUGR	84	47		
brain:spleen	non-IUGR	51	80	*P*<0.001	2.6
	IUGR	82	49		
brain:kidney	non-IUGR	53	78	*P*<0.001	2.8
	IUGR	83	48		

The numbers of fetuses with and without intrauterine growth retardation (IUGR) are presented for low and high birth weight (BW) gilts based on different brain:organ weight ratios (defined as fetuses with brain:organ weight ratios greater than +1 SD from mean (IUGR) or fetuses with brain:organ weight ratios less than -1 SD from mean (non-IUGR)). As weights could not be accurately measured in dead fetuses, this data table is limited to live fetuses (VIA, MEC) only.

### Assessment of PRRSv severity in low and high BW gilts

Following challenge, no gilts demonstrated lethargy, depression or clinical signs of respiratory disease including dyspnea or persistent paroxysmal coughing. Reduced daily feed intake was observed in 17 of 54 (31.5%) low BW and 17 of 57 (29.8%) high BW gilts (*P* = 1.0). In 9/17 low BW and 8/17 high BW gilts, reduced feed intake or complete anorexia was observed for two or more consecutive days. Similarly, the presence of fever, defined as a gilt with a rectal temperature exceeding 39.5°C any day after PRRSv inoculation, did not differ statistically between low and high BW gilts (low BW 22/54, high BW 17/57; *P* = 0.24).

The dam's birth weight group also had no significant effect on litter outcome following PRRSv infection, including the number of fetuses in each fetal preservation category or the percent fetal mortality at the gilt-level (low BW 38.0 ±24.0%, high BW 43.9 ±21.3%; *P* = 0.18). Similarly, the PRRS viral load in gilt serum from 0–21 dpi, as well as in gilt and fetal tissues, did not differ significantly between groups (Table S1).

To investigate differences in immune responses of pregnant gilts following PRRSv infection, changes in PBMC subsets in whole blood and cytokine proteins in gilt serum were compared in low and high BW gilts. A massive drop in total WBC was detected in both low and high BW gilts on 2 dpi. All investigated PBMC subsets were affected to varying degrees (Table S2), but only the AUC between D0 and D19 for γδ T cells decreased significantly in high compared to low BW gilts (high BW 20.3 ±8.5, low BW 23.9 ±10.2,; *P* = 0.03). No significant group differences were found in total WBC or the remaining PBMC subsets over time. In both groups, levels of IFNα, CCL2 and IFNγ in serum increased on 2 dpi (Table S3), but no significant BW group differences were found in serum levels of the investigated cytokines over time.

### PRRSv RNA concentrations in IUGR and non-IUGR fetuses

**Figure 1:**
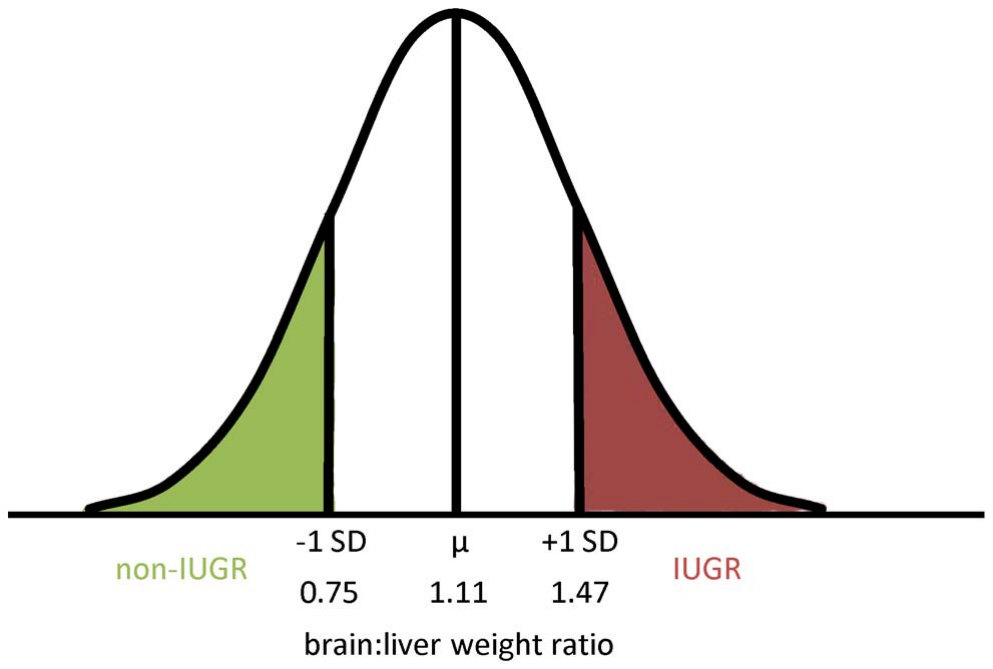
Graphic representation of the distribution of IUGR and non-IUGR fetuses based on brain:liver weight ratios. IUGR fetuses were defined as those with brain:liver weight ratios greater than +1 SD from the mean (ratio> 1.47), while non-IUGR fetuses were those with brain:liver weight ratios less than -1 SD from the mean (ratio <0.75) which representative of the top and bottom 16% of the fetal population.

**Figure 2:**
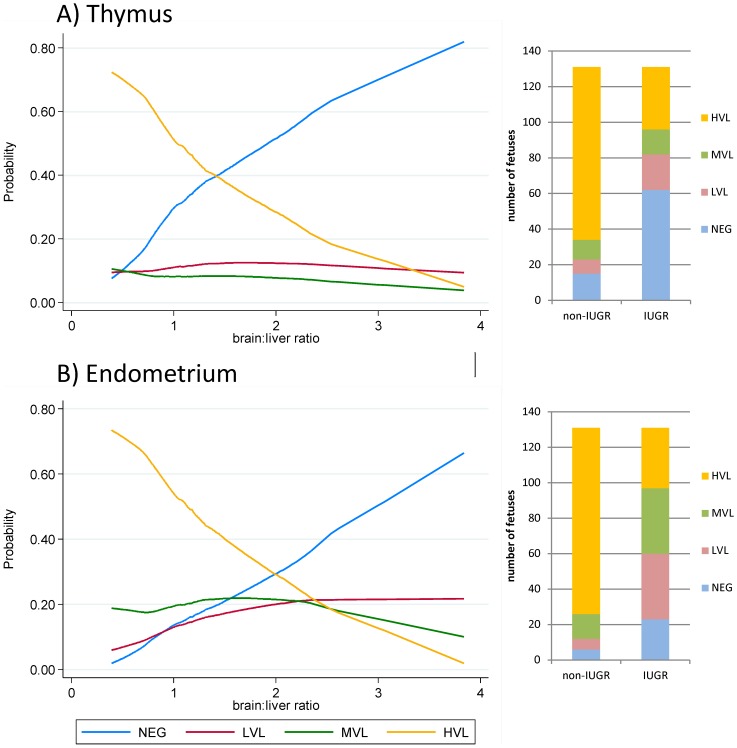
Association of viral load and brain:liver weight ratios of fetuses. *Line charts*: The probability of fetuses falling into various viral load (VL) categories: negative (NEG), low (LVL), medium (MVL), high (HVL) in fetal thymus (A) and endometrium (B) based on brain:liver weight ratio is presented. Fetuses with high brain:liver ratio (IUGR) have increased probability of falling in the NEG viral load category. *Bar charts*: numbers of IUGR and non-IUGR fetuses categorized based on brain:liver weight ratios (IUGR = fetuses with brain:liver weight ratios>1 SD from mean, non-IUGR = fetuses with brain:liver weight ratios <1 SD from mean) are presented for each VL category in fetal thymus (A) and endometrium (B). IUGR = intrauterine growth retardation.

A total of 131 fetuses were categorized as IUGR based on having a brain:organ weight ratio greater than 1 SD from mean and were compared to 131 non-IUGR fetuses with brain:organ weight ratios less than -1 SD from mean (Figure 1). Fetal morphometrics and viral load data for IUGR and non-IUGR fetuses based on brain:liver ratios are summarized in Table 4 along with covariates included in the statistical models. IUGR fetuses had significantly lower PRRSv RNA concentration in fetal thymus and endometrium compared to non-IUGR fetuses (*P*<0.001 for both). This unexpected result was confirmed using a proportional odds model that estimated the probability of fetuses falling into various viral load categories (NEG, LVL, MVL, HVL) based on their brain:organ weight ratios. This analysis included all live fetuses and confirmed that as brain:organ ratio increased, the probability of a fetus being NEG in fetal thymus and endometrium increased, whereas the probability of falling into the HVL category decreased. Figure 2 presents representative data for brain:liver weight ratios. All brain:organ ratios gave similar results with the exception of brain:lung (Tables S4–S7).

**Table 4:** Morphometrics and viral load in IUGR and non-IUGR fetuses categorized based on extreme brain:liver weight ratio.

	Mean (SD)		*P* (β)					
	non IUGR (n = 131)	IUGR (n = 131)	IUGR	Sex	LS	Preservation	VL thymus	LoHi BW
weight fetus	1166 (228)	647 (172)	<0.001 (−479.0)	ns	<0.001 (−20.9)	<0.001 (−112.7)	ns	ns
weight brain	24.9 (2.9)	24.4 (2.6)	ns	0.009 (0.4)	0.007 (−0.1)	<0.001 (−2.0)	<0.001 (−0.1)	ns
weight liver	40.3 (7.9)	13.9 (3.0)	<0.001 (−23.1)	ns	<0.001 (−0.5)	ns	0.018 (0.3)	ns
weight lung	32.6 (8.7)	18.8 (5.5)	<0.001 (−15.3)	ns	<0.001 (−0.7)	<0.001 (−5.2)	<0.001 (−0.5)	ns
weight heart	9.6 (1.8)	5.4 (1.4)	<0.001 (−3.8)	0.043 (0.4)	<0.001 (−0.2)	0.037 (−0.5)	ns	ns
weight spleen	2.1 (0.7)	1.0 (0.4)	<0.001 (−0.7)	0.022 (0.1)	<0.001 (−0.1)	ns	0.001 (0.04)	ns
weight kidney	12.2 (3.1)	6.2 (2.4)	<0.001 (−4.2)	ns	<0.001 (−0.2)	ns	<0.001 (0.2)	ns
brain:liver	0.6 (0.1)	1.8 (0.4)	<0.001 (1.1)	ns	0.027 (0.01)	ns	ns	ns
brain:lung	0.8 (0.2)	1.4 (0.5)	<0.001 (0.6)	ns	0.032 (0.02)	ns	0.005 (0.02)	ns
brain:heart	2.7 (0.5)	4.8 (1.1)	<0.001 (1.8)	ns	0.003 (0.05)	ns	0.031 (−0.04)	ns
brain:spleen	13.3 (4.1)	28.9 (11.6)	<0.001 (11.3)	ns	0.003 (0.5)	ns	0.003 (−0.5)	ns
brain:kidney	2.2 (1.1)	4.5 (2.2)	<0.001 (1.9)	ns	ns	ns	0.001 (−0.1)	ns
CRL	29.3 (2.3)	25.3 (2.3)	<0.001 (−3.9)	ns	0.002 (−0.2)	0.001 (−1.2)	ns	ns
VL thymus*	5.6 (2.7)	2.4 (2.9)	<0.001 (−1.8)	ns	ns	<0.001 (2.1)	not tested	ns
VL endometrium*	5.3 (1.7)	2.9 (2.1)	<0.001 (−1.6)	ns	ns	<0.001 (1.4)	not tested	ns

*Mean log_10_ copies per mg tissue.

*Left columns*: Means (SD) of fetal weight (g), fetal organ weights (g), brain:organ weight ratios, crown-rump-length (CRL, cm), and viral load (VL) in fetal thymus and endometrium (log_10_ copies/mg) are presented for IUGR and non-IUGR fetuses categorized based on brain:liver weight ratios. IUGR fetuses have brain:liver weight ratios greater than +1 SD from mean, non-IUGR fetuses have brain:liver weight ratios less than -1 SD from mean. *Right columns: P* values and beta coefficients (β) obtained by two-level, linear, mixed-effects regression models are presented showing differences between IUGR and non-IUGR fetuses after controlling for covariates possibly influencing fetal weight: sex: 0 = female, 1 = male; LS: effect of a unit increase in litter size (fetal number); Preservation: fetal preservation at termination 0 = viable, 1 = meconium stained; VL thymus = effect of a unit increase in PRRSv RNA concentration (log_10_ target copies/mg) in fetal thymus collected at termination; LoHi BW: 0 = low BW dam, 1 = high BW dam; ns = not significant (*P*>0.05).

### Comparison of CRL across fetal preservation category

**Figure 3:**
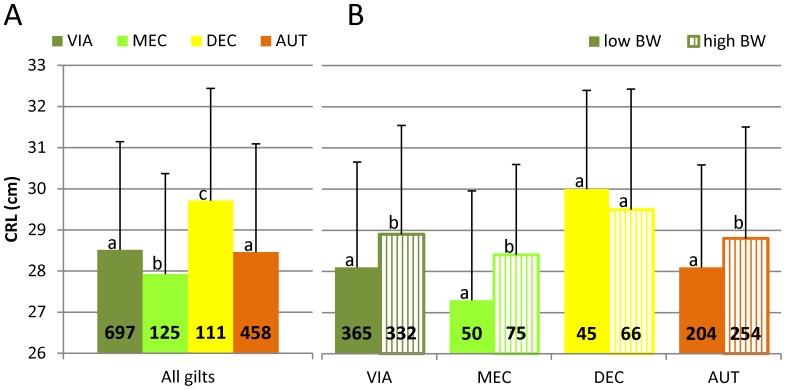
Comparison of fetal crown-rump-length across preservation categories. The mean crown-rump-length (CRL, cm) is presented for fetuses across preservation categories at 21 days after PRRSv inoculation (gestation day 106 ±1). A) All gilts regardless of birth weight; B) Low (solid bars) and high (lined bars) BW gilts. Bars represent standard deviation; values indicate the number of fetuses within each preservation category; letters indicate significant differences (*P*<0.05) between preservation categories (A) or between low and high BW gilts within one preservation category (B). Viable (VIA) and meconium stained (MEC) fetuses were alive at termination, while decomposed (DEC) and autolyzed (AUT) fetuses were dead.

Since viral loads in fetal thymus and endometrium were higher in non-IUGR fetuses, we compared the relative sizes of fetuses that died after PRRSv infection with fetuses that survived until termination 21 days post inoculation. The autolytic process influenced fetal weight (DEC and AUT fetuses were swollen and edematous at necropsy). Therefore, we used CRL as a proxy measurement of fetal size. CRL did not significantly differ between VIA and AUT fetuses (Figure 3A), and in fact, was longest in DEC fetuses. CRL, however, was significantly greater in fetuses from high BW gilts (*P*<0.001, β = 0.84) (Figure 3B) and was negatively related to litter size (*P*<0.001, β = −0.24).

## Discussion

This is the first report investigating relationships between dams' BW, IUGR and the susceptibility to PRRSv in a reproductive model. In contrast to human medicine, there are few studies investigating the prenatal programming of disease susceptibility in pigs. In a recent study, clinical signs, immune responses and pathologic lesions were compared between low and high BW piglets experimentally infected with influenza virus (A/swine/Texas/4199-2/98 H3N2). Although no significant differences could be detected in clinical signs, viral shedding or cytokine production, high BW piglets showed more severe lung lesions, which was contrary to the authors' overarching hypothesis that the severity of disease is greater in low BW animals [33]. Although in the present study high and low BW gilts did not differ in terms of clinical signs, viral load, cytokine responses and fetal outcome (percent dead per litter), high BW gilts did demonstrate a more severe drop in absolute numbers of γδ T cells after experimental infection with PRRSv. This finding was also contrary to our overarching hypothesis that PRRS would be more severe in low BW gilts.

γδ T cells possess features of both innate and adaptive immunity and represent a major T cell population in swine and other ungulates [34], [35]. Reports on γδ T cell responses in PRRSv infected pigs are scarce, but we recently demonstrated that absolute numbers of γδ T cells were negatively associated with viral load in serum from 0–21 days post inoculation (Ladinig, unpublished data). Therefore, γδ T cells may be relevant in resistance or tolerance to PRRSv through natural selection. Even though γδ T cells dropped more severely in high BW gilts, it did not appear to adversely impact litter outcome, thus the relevance of this finding in the reproductive model remains elusive. In a recent study, it was shown that γδ T cell populations were significantly decreased in the lungs, tonsils, and iliac lymph nodes from VR2332 infected pigs at 30 dpi, 60 dpi or both. In lung tissue this drop coincided with an increase of IFNγ producing cells at 30 dpi [36]. In the present study, there was no relationship between IFNγ levels and absolute numbers of γδ T cells which might be explained by the fact that pregnant gilts rather than nursery pigs were used, gilts were terminated at 21 dpi, γδ T cells were quantified in blood not tissues, and a much larger sample size was used.

As described in humans [4], [5], our data clearly demonstrated a transgenerational effect of BW in pigs. Fetuses from low BW gilts had significantly decreased body weight compared to fetuses from high BW gilts. Moreover, low BW gilts had a disproportionately higher number of IUGR fetuses. These results are consistent with evidence of transgenerational trends in litter BW based on the analysis of an extensive dataset from a commercial breeding farm (Patterson *et al.*, unpublished data). Although a repeatable low BW phenotype in mature sows is thought to involve a high ovulation rate and excessive intrauterine crowding as important component traits [37], the observation of the low BW phenotype in the gilt litter, in the absence of any difference in ovulation rate, suggests that other components traits (uterine capacity, placental function) are already exerting effects on BW phenotype in the gilt.

Underlying transgenerational mechanisms proposed in humans include alterations in the uterine or systemic vasculature, programmed changes in maternal metabolic status, or impaired placentation [38]. Data from humans, rats and ruminants indicate that maternal malnutrition during pregnancy induces epigenetic changes in gene expression of fetuses which are transmitted to subsequent generations [39]–[42]. Mechanisms of epigenetic gene regulation include DNA methylation, histone modification and RNA interference, as previously reviewed [43]–[46]. Initial evidence that similar mechanisms mediate metabolic programming of the subsequent litter in pigs has been presented [47]. However, as there were no differences in feed intake between low and high BW gilts during this present experiment, other epigenetic pathways must mediate the transgenerational trend in litter BW phenotype.

Intrauterine growth retardation can be assessed in a number of ways including measurement of body weight, size and shape, placental weight and efficiency, body mass index (BMI) and brain:organ weight ratios. It is important to note that small fetuses are not necessarily growth retarded; IUGR is defined by asymmetrical fetal growth. The exact mechanisms underlying brain sparing are not completely understood and in humans, they are controversial. While some human epidemiologic studies suggest the reallocation of energy and nutrients to organs (e.g. the brain and heart) critical for immediate survival in the event of maternal malnutrition [39], [48], others found little evidence of brain sparing and concluded it might be an artifact determined by the strong inverse correlation between head size and body weight [49], [50]. Similar to our findings, Bauer *et al.* [2] and Town *et al.* [7] described increased brain:organ weight ratios in newborn piglets or fetuses affected by IUGR. In the present study, fetuses with extreme brain:organ weight ratios were defined as IUGR. The finding that viral load in fetal thymus and in the endometrium was significantly lower in IUGR fetuses was unexpected and is contrary to our hypothesis.

The relationship between viral load and brain:organ weight ratio was consistent across all fetal organs investigated with the exception of lung. The fetal lung contains varying amounts of amniotic fluid, and unfortunately, lungs were not thoroughly drained prior to being weighed in this study. While the population of IUGR fetuses used in the present analyses was similar across all brain:organ weight ratios investigated, the population of non-IUGR fetuses differed in the analysis of brain:lung weight ratio compared to all other brain:organ weight ratios. For this reason, we believe that lung weight is not reliable for the classification of IUGR in fetuses.

Unfortunately, only weight data from fetuses that were alive at termination could be included in the brain sparing analysis because autolysis altered fetal and organ weight. Moreover, our assayed viral levels were significantly lower in dead compared to live fetuses, most likely due to viral RNA degradation over time [25]. Given that PRRSv concentration was higher in non-IUGR fetuses, we were interested in assessing if large fetuses were at greater risk of death. Crown rump length, a measurement that is unaffected by autolysis was used to compare the relative size of fetuses across preservation categories. Fetal pigs grow rapidly during the third trimester. Previous studies have shown the CRL increases 2.7 – 3.7 mm per day between gestation day 80 and 100 in crossbred gilts [51]. Breed differences may exist; CRL growth was reported to be 1.9 mm per day in Meishan fetuses and 2.3 mm per day in Yorkshire fetuses between gestation day 90 and 110 [52]. Given the advanced stage of autolysis in AUT fetuses which had discoloured and generally dark brown skin and liquefied internal organs, we hypothesize that AUT fetuses were dead for at least 1 week prior to termination. It was therefore expected that dead fetuses, particularly AUT, would have CRL at least 1.0 to 1.5 cm shorter than live fetuses (VIA and MEC) that had had one additional week of fetal growth. Surprisingly, no difference in CRL was detected. These results add further support to the conclusion that larger fetuses are more susceptible to transplacental PRRSv infection. We believe there are two possible explanations. Firstly, large, non-IUGR fetuses have larger placentae [53]. If transplacental migration of PRRSv or fetal death involve mechanisms at the uteroplacental interface, it is reasonable that larger placentae would result in greater opportunity for transplacental infection or fetal death. This is supported by the fact that virus replication in endometrium and placenta precedes fetal infection and that the number of sialoadhesin positive (Sn^+^)/CD163^+^ macrophages in endometrium and placenta plays a role in fetal death [29], [30], [54]. Secondly, IUGR is associated with persistent changes in tissue structure and functionality [55]. This may in turn affect critical pathways influencing the rate of viral replication. In fact, DNA methylation and histone modification are reported to play important roles in virus life cycles and replication in humans [56].

In conclusion, viral load and litter outcome including the percentage of dead fetuses per litter did not differ between low and high BW gilts. However, high BW gilts showed a more severe post-inoculation drop in γδ T cells over time. The results of the present study fail to provide substantive evidence that the severity of PRRS is related to the birth weight of the dam. Fetal weight, however, was significantly lower and the number of fetuses with IUGR significantly higher in low BW compared to high BW gilts of the same genetic source, confirming BW is a transgenerational trait in pigs. Therefore, BW is a very valuable trait to include in genetic selection programs for replacement females. The most remarkable finding of this study was that fetuses with IUGR had lower PRRS viral loads in both fetal thymus and endometrium. Together with the fact that the CRL did not significantly differ between fetuses that died at least one week prior to termination and fetuses that survived until termination, this provides evidence that bigger fetuses are more susceptible to transplacental PRRSv infection. Further experiments are required to confirm and elucidate mechanisms associated with this novel and industry relevant finding.

## Supporting Information

Figure S1 **Distribution of fetuses across viral load category.** Numbers of fetuses falling into each viral load (VL) category (negative (NEG), low (LVL), medium (MVL), high (HVL)) in fetal thymus (A) and endometrium (B) are presented for all fetuses combined, and for fetuses of each preservation category (VIA = viable fetuses; MEC = meconium stained fetuses; DEC = decomposed fetuses; AUT = autolyzed fetuses). Mean VL ±SD(log_10_ copies/mg tissue) are indicated for positive categories.
(JPG)

Table S1 **Concentration of PRRSv RNA in gilt serum over time, gilt and fetal tissues from low and high birth weight gilts.** Mean (SD) PRRSv RNA concentrations are presented for gilt serum over time (AUC) and gilt tissues and fetal tissues (log_10_ copies/mg) from low and high BW gilts. BW = birth weight, LN = lymph node, AUC = serum viral load (log_10_ copies/µL over 21 days).
(DOCX)

Table S2 **Mean numbers (SD) of white blood cells from low and high birth weight gilts.** Absolute numbers (mean cells x 10^9^/L, SD) of total white blood cell counts (WBC), myeloid cells, NK cells, B cells, total T cells, γδ T cells, T helper cells and cytolytic T cells (CTL) are presented from 54 low and 57 high BW gilts. dpi = days post-inoculation, BW = birth weight, AUC = area under curve from 0–19 dpi.
(DOCX)

Table S3 **Mean cytokine levels (SD) in serum from low and high birth weight gilts following PRRSv inoculation.** Mean cytokine (SD) levels (pg/ml) in serum are presented for the 8 analysed cytokines from 54 low and 57 high BW gilts. dpi = days post-inoculation, BW = birth weight, AUC = area under curve from 0–21 dpi, IL = interleukin, CCL = chemokine ligand, IFN = interferon.
(DOCX)

Table S4 **Morphometrics and viral load in IUGR and non-IUGR fetuses categorized based on extreme brain:lung weight ratios.** *Mean log_10_ copies per mg tissue. *Left columns*: Means (SD) of fetal weight (g), fetal organ weights (g), brain:organ weight ratios, crown-rump-length (CRL, cm), and viral load (VL) in fetal thymus and endometrium (log_10_ copies/mg) are presented for IUGR and non-IUGR fetuses categorized based on brain:lung weight ratios. IUGR fetuses have brain:lung weight ratios greater than +1SD from mean, non-IUGR fetuses have brain:lung weight ratios less than -1 SD from mean. *Right columns*: *P*-values and beta coefficients (β) obtained by two-level, linear, mixed-effects regression models are presented showing differences between IUGR and non-IUGR fetuses after controlling for covariates possibly influencing fetal weight: sex: 0 = female, 1 = male; LS: effect of a unit increase in litter size (fetal number); Preservation: fetal preservation at termination 0 = viable, 1 = meconium stained; VL_thymus = effect of a unit increase in PRRSv RNA concentration (log_10_ target copies/mg) in fetal thymus collected at termination; LoHi BW: not significant; ns = not significant (*P* >0.05).
(DOCX)

Table S5 **Morphometrics and viral load in IUGR and non-IUGR fetuses categorized based on extreme brain:heart weight ratios.** *Mean log_10_ copies per mg tissue. *Left columns*: Means (SD) of fetal weight (g), fetal organ weights (g), brain:organ weight ratios, crown-rump-length (CRL, cm), and viral load (VL) in fetal thymus and endometrium (log_10_ copies/mg) are presented for IUGR and non-IUGR fetuses categorized based on brain:heart weight ratios. IUGR fetuses have brain:heart weight ratios greater than +1SD from mean, non-IUGR fetuses have brain:heart weight ratios less than -1 SD from mean. *Right columns*: *P*-values and beta coefficients (β) obtained by two-level, linear, mixed-effects regression models are presented showing differences between IUGR and non-IUGR fetuses after accounting for covariates possibly influencing fetal weight: sex: 0 = female, 1 = male; LS: effect of a unit increase in litter size (fetal number); Preservation: fetal preservation at termination 0 = viable, 1 = meconium stained; VL_thymus = effect of a unit increase in PRRSv RNA concentration (log_10_ target copies/mg) in fetal thymus collected at termination; LoHi BW: 0 = low BW dam, 1 = high BW dam; ns = not significant (*P* >0.05).
(DOCX)

Table S6 **Morphometrics and viral load in IUGR and non-IUGR fetuses categorized based on extreme brain:spleen weight ratios.** *Mean log_10_ copies per mg tissue. *Left columns*: Means (SD) of fetal weight (g), fetal organ weights (g), brain:organ weight ratios, crown-rump-length (CRL, cm), and viral load (VL) in fetal thymus and endometrium (log_10_ copies/mg) are presented for IUGR and non-IUGR fetuses categorized based on brain:spleen weight ratios. IUGR fetuses have brain:spleen weight ratios greater than +1 SD from mean, non-IUGR fetuses have brain:spleen weight ratios less than -1 SD from mean. *Right columns*: *P*-values and beta coefficients (β) obtained by two-level, linear, mixed-effects regression models are presented showing differences between IUGR and non-IUGR fetuses after controlling for covariates possibly influencing fetal weight: sex: 0 = female, 1 = male; LS: effect of a unit increase in litter size (fetal number); Preservation: fetal preservation at termination 0 = viable, 1 = meconium stained; VL_thymus = effect of a unit increase in PRRSv RNA concentration (log_10_ target copies/mg) in fetal thymus collected at termination; LoHi BW: not significant; ns = not significant (*P* >0.05).
(DOCX)

Table S7 **Morphometrics and viral load in IUGR and non-IUGR fetuses categorized based on extreme brain:kidney weight ratios.** *Mean log_10_ copies per mg tissue. *Left columns*: Means (SD) of fetal weight (g), fetal organ weights (g), brain:organ weight ratios, crown-rump-length (CRL, cm), and viral load (VL) in fetal thymus and endometrium (log_10_ copies/mg) are presented for IUGR and non-IUGR fetuses categorized based on brain:kidney weight ratios. IUGR fetuses have brain:kidney weight ratios greater than +1 SD from mean, non-IUGR fetuses have brain:kidney weight ratios less than -1 SD from mean. *Right columns*: *P*-values and beta coefficients (β) obtained by two-level, linear, mixed-effects regression models are presented showing differences between IUGR and non-IUGR fetuses after accounting for covariates possibly influencing fetal weight: sex: 0 = female, 1 = male; LS: effect of a unit increase in litter size (fetal number); Preservation: fetal preservation at termination 0 = viable, 1 = meconium stained; VL_thymus = effect of a unit increase in PRRSv RNA concentration (log_10_ target copies/mg) in fetal thymus collected at termination; LoHi BW: not significant; ns = not significant (*P*>0.05).
(DOCX)
