# Supplementary figures and images for: Birth Weight, Intrauterine Growth Retardation and Fetal Susceptibility to Porcine Reproductive and Respiratory Syndrome Virus
Source: PLoS One. 2014 Oct 2;9(10):e109541. doi: 10.1371/journal.pone.0109541 (PMC4183575; doi:10.1371/journal.pone.0109541)

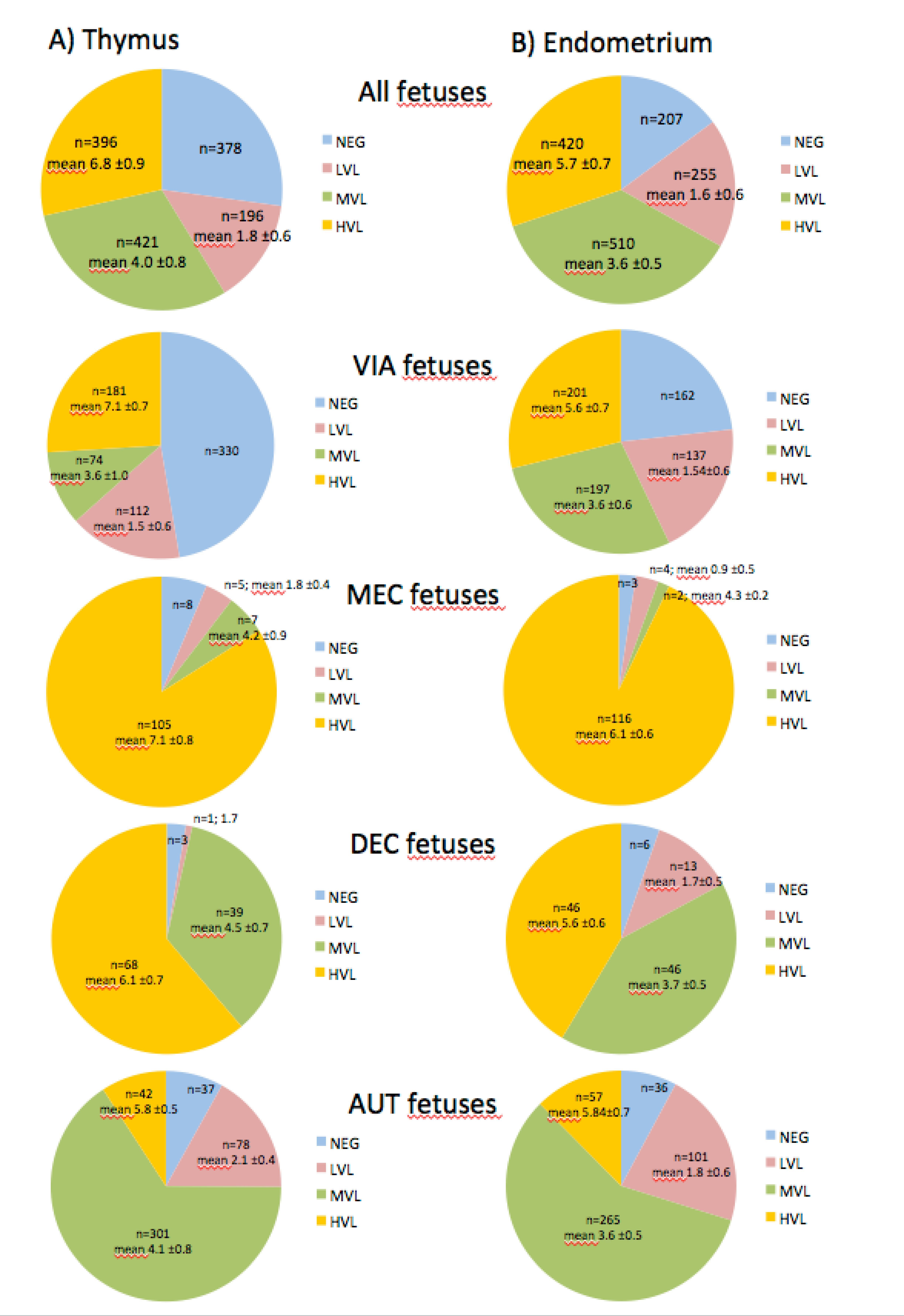

Supplement: Figure S1 — Distribution of fetuses across viral load category. Numbers of fetuses falling into each viral load (VL) category (negative (NEG), low (LVL), medium (MVL), high (HVL)) in fetal thymus (A) and endometrium (B) are presented for all fetuses combined, and for fetuses of each preservation category (VIA = viable fetuses; MEC = meconium stained fetuses; DEC = decomposed fetuses; AUT = autolyzed fetuses). Mean VL ±SD(log10 copies/mg tissue) are indicated for positive categories. (JPG) [file pone.0109541.s001.jpg]
