# Supplementary material for: Birth Weight, Intrauterine Growth Retardation and Fetal Susceptibility to Porcine Reproductive and Respiratory Syndrome Virus
Source: PLoS One. 2014 Oct 2;9(10):e109541. doi: 10.1371/journal.pone.0109541 (PMC4183575; doi:10.1371/journal.pone.0109541)
Supplement: Table S1 — Concentration of PRRSv RNA in gilt serum over time, gilt and fetal tissues from low and high birth weight gilts. Mean (SD) PRRSv RNA concentrations are presented for gilt serum over time (AUC) and gilt tissues and fetal tissues (log10 copies/mg) from low and high BW gilts. BW = birth weight, LN = lymph node, AUC = serum viral load (log10 copies/µL over 21 days). (DOCX) [file pone.0109541.s002.docx]

Supplementary Table 1: Concentration of PRRSv RNA in gilt serum over time, gilt and fetal tissues from low and high birth weight gilts

|  | low BW (n=54) | high BW (n=57) | *P* value |
| --- | --- | --- | --- |
| gilt serum_AUC | 55.6 (9.8) | 56.2 (9.6) | 0.75 |
| gilt lung | 3.3 (1.6) | 3.0 (1.6) | 0.41 |
| gilt tonsil | 5.5 (1.1) | 5.5 (0.9) | 0.72 |
| gilt reproductive LN | 5.9 (0.6) | 5.7 (0.9) | 0.12 |
| gilt tracheobronchial LN | 4.7 (1.1) | 4.8 (0.8) | 0.55 |
| fetal thymus | 3.2 (2.7) | 3.6 (2.7) | 0.63 |
| endometrium | 3.3 (2.0) | 3.3 (2.1) | 0.57 |
