# Supplementary material for: Birth Weight, Intrauterine Growth Retardation and Fetal Susceptibility to Porcine Reproductive and Respiratory Syndrome Virus
Source: PLoS One. 2014 Oct 2;9(10):e109541. doi: 10.1371/journal.pone.0109541 (PMC4183575; doi:10.1371/journal.pone.0109541)
Supplement: Table S2 — Mean numbers (SD) of white blood cells from low and high birth weight gilts. Absolute numbers (mean cells x 109/L, SD) of total white blood cell counts (WBC), myeloid cells, NK cells, B cells, total T cells, γδ T cells, T helper cells and cytolytic T cells (CTL) are presented from 54 low and 57 high BW gilts. dpi = days post-inoculation, BW = birth weight, AUC = area under curve from 0–19 dpi. (DOCX) [file pone.0109541.s003.docx]

Supplementary Table 2: Mean numbers (SD) of white blood cells from low and high birth weight gilts following PRRSv inoculation

|  | dpi | low BW (n=54) | high BW (n=57) |
| --- | --- | --- | --- |
| Total WBC | 0 | 11.0 (2.0) | 11.1 (1.8) |
|  | 2 | 6.0 (2.3) | 6.2 (2.4) |
|  | 6 | 8.4 (1.7) | 8.4 (1.8) |
|  | 19 | 11.5 (2.9) | 11.8 (3.2) |
|  | AUC | 175.1 (31.8) | 177.5 (30.3) |
| Myeloid cells | 0 | 0.8 (0.2) | 0.8 (0.2) |
|  | 2 | 0.6 (0.3) | 0.6 (0.2) |
|  | 6 | 0.7 (0.2) | 0.7 (0.2) |
|  | 19 | 0.9 (0.4) | 1.0 (0.4) |
|  | AUC | 14.6 (4.0) | 14.9 (4.0) |
| NK cells | 0 | 0.22 (0.12) | 0.21 (0.12) |
|  | 2 | 0.05 (0.03) | 0.05 (0.04) |
|  | 6 | 0.12 (0.07) | 0.11 (0.06) |
|  | 19 | 0.22 (0.16) | 0.22 (0.12) |
|  | AUC | 2.8 (1.6) | 2.8 (1.1) |
| B cells | 0 | 1.1 (0.5) | 1.1 (0.4) |
|  | 2 | 0.7 (0.3) | 0.6 (0.3) |
|  | 6 | 0.6 (0.2) | 0.6 (0.2) |
|  | 19 | 1.0 (0.3) | 1.0 (0.4) |
|  | AUC | 14.3 (4.6) | 14.3 (5.0) |
| T cells | 0 | 5.1 (1.3) | 4.9 (1.2) |
|  | 2 | 2.0 (0.9) | 1.8 (0.8) |
|  | 6 | 4.3 (1.2) | 4.1 (1.2) |
|  | 19 | 4.3 (1.3) | 4.3 (1.2) |
|  | AUC | 75.3 (17.9) | 73.5 (16.7) |
| γδ T cells | 0 | 1.7 (0.7) | 1.5 (0.6) |
|  | 2 | 0.9 (0.6) | 0.8 (0.5) |
|  | 6 | 1.4 (0.6) | 1.1 (0.5) |
|  | 19 | 1.1 (0.6) | 1.0 (0.5) |
|  | AUC | 23.9 (10.2) | 20.3 (8.5) |
| T helper cells | 0 | 1.9 (0.5) | 1.9 (0.4) |
|  | 2 | 0.6 (0.3) | 0.6 (0.2) |
|  | 6 | 1.6 (0.4) | 1.6 (0.4) |
|  | 19 | 1.5 (0.5) | 1.6 (0.4) |
|  | AUC | 26.5 (5.9) | 27.3 (5.7) |
| CTL | 0 | 1.4 (0.4) | 1.4 (0.5) |
|  | 2 | 0.3 (0.2) | 0.3 (0.2) |
|  | 6 | 1.2 (0.4) | 1.2 (0.4) |
|  | 19 | 1.4 (0.5) | 1.5 (0.6) |
|  | AUC | 21.6 (5.78) | 22.0 (7.0) |
