# Supplementary material for: Birth Weight, Intrauterine Growth Retardation and Fetal Susceptibility to Porcine Reproductive and Respiratory Syndrome Virus
Source: PLoS One. 2014 Oct 2;9(10):e109541. doi: 10.1371/journal.pone.0109541 (PMC4183575; doi:10.1371/journal.pone.0109541)
Supplement: Table S3 — Mean cytokine levels (SD) in serum from low and high birth weight gilts following PRRSv inoculation. Mean cytokine (SD) levels (pg/ml) in serum are presented for the 8 analysed cytokines from 54 low and 57 high BW gilts. dpi = days post-inoculation, BW = birth weight, AUC = area under curve from 0–21 dpi, IL = interleukin, CCL = chemokine ligand, IFN = interferon. (DOCX) [file pone.0109541.s004.docx]

Supplementary Table 3: Mean cytokine levels (SD) in serum from low and high birth weight gilts following PRRSv inoculation

|  | dpi | low BW (n=54) | high BW (n=57) |
| --- | --- | --- | --- |
| IL1β | 0 | 48 (128) | 66 (263) |
|  | 2 | 43 (115) | 55 (207) |
|  | 6 | 41 (93) | 60 (260) |
|  | 21 | 71 (140) | 44 (78) |
|  | AUC | 1099 (2169) | 1031 (3747) |
| IL8 | 0 | 105 (114) | 108 (109) |
|  | 2 | 99 (109) | 96 (90) |
|  | 6 | 133 (110) | 143 (101) |
|  | 21 | 125 (131) | 109 (77) |
|  | AUC | 2601 (2283) | 2572 (1635) |
| CCL2 | 0 | 1034 (501) | 1037 (6075) |
|  | 2 | 5699 (2920) | 6466 (5613) |
|  | 6 | 3953 (2625) | 4373 (2654) |
|  | 21 | 1503 (1254) | 1252 (855) |
|  | AUC | 66956 (32776) | 71376 (40273) |
| IFNα | 0 | 7 (18) | 11 (49) |
|  | 2 | 399 (189) | 499 (314) |
|  | 6 | 78 (60) | 81 (60) |
|  | 21 | 9 (13) | 7 (10) |
|  | AUC | 2012 (954) | 2332 (1268) |
| IFNγ | 0 | 79 (244) | 65 (126) |
|  | 2 | 129 (211) | 115 (130) |
|  | 6 | 75 (173) | 84 (173) |
|  | 21 | 47 (85) | 43 (83) |
|  | AUC | 1532 (2958) | 1533 (2622) |
| IL12 | 0 | 134 (160) | 161 (461) |
|  | 2 | 101 (122) | 95 (160) |
|  | 6 | 115 (125) | 146 (451) |
|  | 21 | 399 (1039) | 183 (177) |
|  | AUC | 4518 (8473) | 3205 (5681) |
| IL4 | 0 | 9 (23) | 13 (25) |
|  | 2 | 7 (21) | 9 (17) |
|  | 6 | 7 (24) | 10 (22) |
|  | 21 | 28 (93) | 20 (54) |
|  | AUC | 306 (794) | 278 (643) |
| IL10 | 0 | 11 (22) | 12 (25) |
|  | 2 | 11 (24) | 9 (17) |
|  | 6 | 10 (21) | 12 (21) |
|  | 21 | 20 (28) | 22 (44) |
|  | AUC | 294 (445) | 325 (489) |
