# Supplementary material for: Birth Weight, Intrauterine Growth Retardation and Fetal Susceptibility to Porcine Reproductive and Respiratory Syndrome Virus
Source: PLoS One. 2014 Oct 2;9(10):e109541. doi: 10.1371/journal.pone.0109541 (PMC4183575; doi:10.1371/journal.pone.0109541)
Supplement: Table S4 — Morphometrics and viral load in IUGR and non-IUGR fetuses categorized based on extreme brain:lung weight ratios. *Mean log10 copies per mg tissue. Left columns: Means (SD) of fetal weight (g), fetal organ weights (g), brain:organ weight ratios, crown-rump-length (CRL, cm), and viral load (VL) in fetal thymus and endometrium (log10 copies/mg) are presented for IUGR and non-IUGR fetuses categorized based on brain:lung weight ratios. IUGR fetuses have brain:lung weight ratios greater than +1SD from mean, non-IUGR fetuses have brain:lung weight ratios less than -1 SD from mean. Right columns: P-values and beta coefficients (β) obtained by two-level, linear, mixed-effects regression models are presented showing differences between IUGR and non-IUGR fetuses after controlling for covariates possibly influencing fetal weight: sex: 0 = female, 1 = male; LS: effect of a unit increase in litter size (fetal number); Preservation: fetal preservation at termination 0 = viable, 1 = meconium stained; VL_thymus = effect of a unit increase in PRRSv RNA concentration (log10 target copies/mg) in fetal thymus collected at termination; LoHi BW: not significant; ns = not significant (P >0.05). (DOCX) [file pone.0109541.s005.docx]

Supplementary Table 4: Morphometrics and viral load in IUGR and non-IUGR fetuses categorized based on extreme brain:lung weight ratios

|  | Mean (SD) | | *P* (β) | | | | | |
| --- | --- | --- | --- | --- | --- | --- | --- | --- |
|  | non IUGR (n=131) | IUGR (n=131) | IUGR | sex | litter size | fet_pres | PCR_fet_thy |  |
| weight fetus | 1244 (187) | 671 (208) | <0.001 (-505.4) | ns | <0.001 (-16.2) | 0.006 (-87.8) | 0.010 (10.0) |  |
| weight brain | 26.9 (3.0) | 24.4 (2.7) | <0.001 (-2.2) | ns | ns | <0.001 (-2.3) | ns |  |
| weight liver | 33.9 (8.6) | 16.7 (6.0) | <0.001 (-15.0) | 0.013 (1.8) | <0.001 (-0.6) | 0.001 (3.5) | <0.001 (0.7) |  |
| weight lung | 41.1 (6.0) | 16.5 (3.6) | <0.001 (-23.3) | 0.023 (1.3) | 0.026 (-0.2) | <0.001 (-3.2) | ns |  |
| weight heart | 10.0 (1.7) | 5.7 (1.8) | <0.001 (-3.7) | ns | 0.001 (-0.1) | ns | 0.026 (0.1) |  |
| weight spleen | 1.9 (0.7) | 1.1 (0.5) | <0.001 (-0.7) | 0.014 (0.2) | <0.001 (-0.05) | ns | <0.001 (0.1) |  |
| weight kidney | 11.7 (2.9) | 7.0 (3.1) | <0.001 (-3.8) | ns | <0.001 (-0.2) | ns | <0.001 (0.2) |  |
| brain:liver | 0.8 (0.2) | 1.6 (0.5) | <0.001 (0.7) | ns | <0.001 (0.02) | <0.001 (-0.2) | <0.001 (-0.03) |  |
| brain:lung | 0.7 (0.1) | 1.6 (0.4) | <0.001 (0.9) | ns | ns | ns | ns |  |
| brain:heart | 2.8 (0.5) | 4.6 (1.3) | <0.001 (1.7) | ns | 0.001 (0.1) | ns | <0.001 (-0.1) |  |
| brain:spleen | 15.4 (4.3) | 26.8 (11.9) | <0.001 (9.6) | ns | 0.002 (0.5) | ns | <0.001 (-0.7) |  |
| brain:kidney | 2.5 (1.1) | 4.1 (1.5) | <0.001 (1.3) | ns | 0.003 (0.1) | ns | <0.001 (-0.1) |  |
| CRL | 30.4 (2.1) | 25.3 (2.5) | <0.001 (-4.2) | ns | <0.001 (-0.2) | 0.012 (-0.90) | ns |  |
| VL thymus* | 2.3 (3.0) | 3.5 (3.2) | <0.001 (1.4) | ns | ns | <0.001 (2.3) | not tested |  |
| VL endometrium* | 2.9 (2.4) | 3.5 (2.2) | 0.019 (0.7) | ns | ns | <0.001 (1.9) | not tested |  |
