# Supplementary material for: Birth Weight, Intrauterine Growth Retardation and Fetal Susceptibility to Porcine Reproductive and Respiratory Syndrome Virus
Source: PLoS One. 2014 Oct 2;9(10):e109541. doi: 10.1371/journal.pone.0109541 (PMC4183575; doi:10.1371/journal.pone.0109541)
Supplement: Table S5 — Morphometrics and viral load in IUGR and non-IUGR fetuses categorized based on extreme brain:heart weight ratios. *Mean log10 copies per mg tissue. Left columns: Means (SD) of fetal weight (g), fetal organ weights (g), brain:organ weight ratios, crown-rump-length (CRL, cm), and viral load (VL) in fetal thymus and endometrium (log10 copies/mg) are presented for IUGR and non-IUGR fetuses categorized based on brain:heart weight ratios. IUGR fetuses have brain:heart weight ratios greater than +1SD from mean, non-IUGR fetuses have brain:heart weight ratios less than -1 SD from mean. Right columns: P-values and beta coefficients (β) obtained by two-level, linear, mixed-effects regression models are presented showing differences between IUGR and non-IUGR fetuses after accounting for covariates possibly influencing fetal weight: sex: 0 = female, 1 = male; LS: effect of a unit increase in litter size (fetal number); Preservation: fetal preservation at termination 0 = viable, 1 = meconium stained; VL_thymus = effect of a unit increase in PRRSv RNA concentration (log10 target copies/mg) in fetal thymus collected at termination; LoHi BW: 0 = low BW dam, 1 = high BW dam; ns = not significant (P >0.05). (DOCX) [file pone.0109541.s006.docx]

Supplementary Table 5: Morphometrics and viral load in IUGR and non-IUGR fetuses categorized based on extreme brain:heart weight ratios

|  | Mean (SD) | | *P* (β) | | | | | |
| --- | --- | --- | --- | --- | --- | --- | --- | --- |
|  | non IUGR (n=131) | IUGR (n=131) | IUGR | Sex | LS | Preservation | VLthymus | LoHi BW |
| weight fetus | 1260 (207) | 646 (162) | <0.001 (-567.4) | ns | <0.001 (-15.2) | <0.001 (-109.3) | ns | 0.028 (64.5) |
| weight brain | 26.2 (3.1) | 24.9 (2.8) | <0.001 (-2.1) | ns | ns | <0.001 (-2.6) | 0.014 (-0.1) | ns |
| weight liver | 37.3 (9.6) | 16.3 (4.7) | <0.001 (-17.4) | ns | 0.003 (-0.5) | ns | <0.001 (0.5) | ns |
| weight lung | 35.2 (8.4) | 19.5 (6.3) | <0.001 (-16.2) | ns | 0.011 (-0.4) | 0.001 (-4.1) | <0.001 (-0.7) | ns |
| weight heart | 11.0 (1.4) | 5.0 (1.0) | <0.001 (-5.9) | ns | <0.001 (-0.1) | <0.001 (-0.9) | ns | ns |
| weight spleen | 2.1 (0.8) | 0.9 (0.3) | <0.001 (-0.9) | ns | 0.001 (-0.05) | ns | 0.002 (0.04) | ns |
| weight kidney | 12.8 (3.5) | 6.0 (2.0) | <0.001 (-5.7) | ns | 0.021 (-0.1) | ns | <0.001 (0.2) | ns |
| brain:liver | 0.8 (0.3) | 1.6 (0.4) | <0.001 (0.7) | ns | 0.008 (0.02) | 0.003 (-0.2) | ns | ns |
| brain:lung | 0.8 (0.2) | 1.4 (0.5) | <0.001 (0.7) | ns | ns | ns | 0.001 (0.03) | ns |
| brain:heart | 2.4 (0.2) | 5.1 (0.9) | <0.001 (2.6) | ns | 0.008 (0.03) | ns | ns | ns |
| brain:spleen | 13.6 (5.2) | 29.5 (10.8) | <0.001 (13.1) | ns | 0.006 (0.5) | ns | ns | ns |
| brain:kidney | 2.2 (1.1) | 4.5 (1.3) | <0.001 (1.9) | ns | 0.043 (0.05) | ns | <0.001 (-0.1) | ns |
| CRL | 30.2 (2.2) | 25.1 (2.2) | <0.001 (-5.0) | ns | ns | <0.001 (-1.3) | ns | ns |
| VL thymus* | 4.1 (3.3) | 2.0 (2.8) | 0.027 (-1.0) | 0.037 (0.7) | ns | <0.001 (2.5) | not tested | ns |
| VL endometrium* | 4.1 (2.4) | 2.7 (2.2) | 0.027 (-0.7) | ns | ns | <0.001 (2.1) | not tested | ns |
