# Supplementary material for: Birth Weight, Intrauterine Growth Retardation and Fetal Susceptibility to Porcine Reproductive and Respiratory Syndrome Virus
Source: PLoS One. 2014 Oct 2;9(10):e109541. doi: 10.1371/journal.pone.0109541 (PMC4183575; doi:10.1371/journal.pone.0109541)
Supplement: Table S7 — Morphometrics and viral load in IUGR and non-IUGR fetuses categorized based on extreme brain:kidney weight ratios. *Mean log10 copies per mg tissue. Left columns: Means (SD) of fetal weight (g), fetal organ weights (g), brain:organ weight ratios, crown-rump-length (CRL, cm), and viral load (VL) in fetal thymus and endometrium (log10 copies/mg) are presented for IUGR and non-IUGR fetuses categorized based on brain:kidney weight ratios. IUGR fetuses have brain:kidney weight ratios greater than +1 SD from mean, non-IUGR fetuses have brain:kidney weight ratios less than -1 SD from mean. Right columns: P-values and beta coefficients (β) obtained by two-level, linear, mixed-effects regression models are presented showing differences between IUGR and non-IUGR fetuses after accounting for covariates possibly influencing fetal weight: sex: 0 = female, 1 = male; LS: effect of a unit increase in litter size (fetal number); Preservation: fetal preservation at termination 0 = viable, 1 = meconium stained; VL_thymus = effect of a unit increase in PRRSv RNA concentration (log10 target copies/mg) in fetal thymus collected at termination; LoHi BW: not significant; ns = not significant (P>0.05). (DOCX) [file pone.0109541.s008.docx]

Supplementary Table 7: Morphometrics and viral load in IUGR and non-IUGR fetuses categorized based on extreme brain:kidney weight ratios

|  | Mean (SD) | | *P* (β) | | | | | |
| --- | --- | --- | --- | --- | --- | --- | --- | --- |
|  | non IUGR (n=131) | IUGR (n=131) | IUGR | Sex | LS | Preservation | VLthymus | LoHi BW |
| weight fetus | 1196 (236) | 671 (189) | <0.001 (-444.0) | ns | <0.001 (-23.4) | <0.001 (-137.1) | ns | 0.017 (75.5) |
| weight brain | 25.7 (3.0) | 25.0 (2.7) | 0.011 (-1.2) | 0.034 (0.6) | 0.047 (-0.1) | <0.001 (-2.1) | 0.001 (-0.2) | ns |
| weight liver | 35.6 (10.7) | 16.8 (5.4) | <0.001 (-13.6) | ns | <0.001 (-0.8) | 0.001 (4.4) | ns | ns |
| weight lung | 31.4 (9.1) | 20.6 (6.7) | <0.001 (-11.7) | ns | <0.001 (-0.7) | 0.005 (-3.3) | <0.001 (-1.0) | ns |
| weight heart | 9.7 (2.0) | 5.5 (1.5) | <0.001 (-3.7) | ns | <0.001 (-0.2) | 0.016 (-0.7) | ns | ns |
| weight spleen | 2.1 (0.7) | 1.0 (0.3) | <0.001 (-0.7) | 0.008 (0.2) | <0.001 (-0.05) | ns | 0.007 (0.03) | ns |
| weight kidney | 14.6 (2.7) | 5.2 (1.1) | <0.001 (-9.0) | ns | 0.030 (-0.1) | 0.040 (-0.7) | ns | ns |
| brain:liver | 0.8 (0.3) | 1.6 (0.4) | <0.001 (0.6) | ns | <0.001 (0.03) | 0.001 (-0.2) | ns | 0.029 (-0.1) |
| brain:lung | 0.9 (0.5) | 1.3 (0.4) | <0.001 (0.4) | ns | 0.004 (0.02) | ns | 0.016 (0.02) | ns |
| brain:heart | 2.7 (0.6) | 4.8 (1.1) | <0.001 (1.8) | ns | <0.001 (0.1) | ns | ns | ns |
| brain:spleen | 13.8 (5.0) | 29.0 (10.9) | <0.001 (12.7) | ns | 0.005 (0.5) | ns | ns | ns |
| brain:kidney | 1.8 (0.2) | 5.1 (2.1) | <0.001 (3.3) | ns | ns | ns | ns | ns |
| CRL | 29.6 (2.4) | 25.5 (2.4) | <0.001 (-4.1) | ns | <0.001 (-0.2) | 0.004 (-1.1) | 0.009 (-0.1) | ns |
| VL thymus* | 5.4 (2.9) | 1.5 (2.4) | <0.001 (-3.0) | 0.045 (0.6) | ns | <0.001 (1.6) | not tested | ns |
| VL endometrium* | 4.9 (2.0) | 2.6 (2.1) | <0.001 (-1.7) | 0.013 (0.6) | ns | <0.001 (1.4) | not tested | ns |
